# Multitasking in Older Adults’ Daily Activities: A Preliminary Investigation

**DOI:** 10.1093/geroni/igab046.2653

**Published:** 2021-12-17

**Authors:** Abigail Stephan, Junyan Tian, Lesley Ross

**Affiliations:** 1 Clemson University, Clemson, South Carolina, United States; 2 Penn State University, State College, Pennsylvania, United States

## Abstract

The ability to multitask, defined as conducting two or more activities simultaneously, is important in daily life. The majority of prior work has examined multitasking in laboratory settings. However, less is known about how multitasking in daily activities is related to older adults’ executive functioning and perceptions of aging. The current study investigated these relationships in a sample of 33 older adults aged 65-81 (M=70.0, SD=3.6). Participants were asked to describe activities they did each day and estimate time spent in each activity across fourteen days; multiple activities reported in the same time frame were considered multitasking. Executive function was measured at baseline using the Trail Making Test Part B (TMTB), with higher scores indicating worse performance. Expectations regarding aging were measured at baseline using the Expectations Regarding Aging (ERA-12) survey, with higher scores indicating more positive perceptions. Twenty-seven participants (81.82%) reported at least one instance of multitasking in the fourteen-day period. Participants were divided into three groups based on the median number of reported multitasks: no multitasking (n=6), low multitasking (≤4; n=15), and high multitasking (>4; n=12). Although there were no significant differences within the ANOVA, participants who reported low multitasking trended towards poorer executive function and more positive expectations of aging (M_TMTB=100.28, M_ERA= 64.88) than both no multitasking (M_TMTB=82.12, M_ERA=50.46) and high multitasking groups (M_TMTB=94.90, M_ERA= 54.29). Additional research should investigate these possible relationships in larger samples and explore how covariates, such as gender and age, may moderate possible relationships.

